# The rearranged mitochondrial genome of *Leptopilina boulardi* (Hymenoptera: Figitidae), a parasitoid wasp of *Drosophila*

**DOI:** 10.1590/1678-4685-GMB-2016-0062

**Published:** 2016-09-19

**Authors:** Daniel S. Oliveira, Tiago M.F.F. Gomes, Elgion L.S. Loreto

**Affiliations:** 1 Curso Ciências Biológicas, Universidade Federal de Santa Maria (UFSM), Santa Maria, RS, Brazil.; 2 Departamento de Bioquímica e Biologia Molecular (CCNE), Universidade Federal de Santa Maria (UFSM), Santa Maria, RS, Brazil.

**Keywords:** Mitogenome, Cynipoidea, Leptopilina, parasitic wasp

## Abstract

The partial mitochondrial genome sequence of *Leptopilina boulardi* (Hymenoptera: Figitidae) was characterized. Illumina sequencing was used yielding 35,999,679 reads, from which 102,482 were utilized in the assembly. The length of the sequenced region of this partial mitochondrial genome is 15,417 bp, consisting of 13 protein-coding, two rRNA, and 21tRNA genes (the trnaM failed to be sequenced) and a partial A+T-rich region. All protein-coding genes start with ATN codons. Eleven protein-coding genes presented TAA stop codons, whereas ND6 and COII that presented TA, and T nucleotides, respectively. The gene pattern revealed extensive rearrangements compared to the typical pattern generally observed in insects. These rearrangements involve two protein-coding and two ribosomal genes, along with the 16 tRNA genes. This gene order is different from the pattern described for *Ibalia leucospoides* (Ibaliidae, Cynipoidea), suggesting that this particular gene order can be variable among Cynipoidea superfamily members. A maximum likelihood phylogenetic analysis of the main groups of Apocrita was performed using amino acid sequence of 13 protein-coding genes, showing monophyly for the Cynipoidea superfamily within the Hymenoptera phylogeny.

*Leptopilina boulardi* is a larval parasitoid of Drosophilidae, mainly of the *Drosophila* species. Originally from Africa, today it is almost cosmopolitan, having been found in the Mediterranean area, tropical Africa and the Americas (Allemand *et al.*, 2002). *Drosophila* species, primarily *D. melanogaster,* are centenary model organisms and *L. boulardi* is an ideal *Drosophila* partner for studying the relationship between insect-parasitoids involving ecology, evolutionary, physiology, immunology, and parasitoid viruses (Carton and Nappi, 1997; Varaldi *et al.*, 2006; Dubuffet *et al.*, 2007; Prévost, 2009). Furthermore, Guimarães *et al.* (2003) put forth that this parasitoid wasp has the potential to be used in strategies of integrated pest management (IPM) of frugivorous Diptera, mostly Drosophilidae pests like *Drosophila suzukii* and *Zaprionus indianus. L. boulardi* belongs to the Cynipoidea superfamily, formed by five families of extant species and three other families that are extinct (Ronquist, 1999; Liu *et al.*, 2007; Sharkey *et al.*, 2012). Figitidae is the family which has the largest number of described species within Cynipoidea, with a global diversity estimate of 24,000 species, all being chiefly parasitoids of flies (Buffington *et al.*, 2007).

Complete mitochondrial genomes are useful models for molecular evolution and powerful tools for phylogenetic and population studies. Most animal mitogenomes are about 16 kb in size and contain 37 genes: 13 protein-coding genes, 22 transfer RNA genes (tRNA), and two ribosomal RNA genes (rRNA) (Boore, 1999). Mitochondrial phylogenomics has been used to study relationship among the more basal clades of living beings (Bernt *et al.*, 2013b), but it is also useful for analyses of more inclusive taxa (Ruiz-Trillo *et al.*, 2008). For these studies, genomic sequences are used, as well as the mitochondrial gene order (GO). In insects, gene synteny is a well conserved character, and the arrangement more widely distributed is referred to as “Pancrustacea ancestral Gene Order” (PanGo). However, certain insects, such as Hymenoptera, Psocoptera, Phthiraptera, and others exhibit rearrangements in their GO, and this characteristic can be very informative for phylogenetic analysis (Wei *et al.*, 2010; Babbucci *et al.*, 2014).

So far, in the Cynipoidea superfamily, only *Ibalia leucospoides*, from the Ibaliidae family, has had its mitogenome described (Mao *et al.*, 2015). In the present study, the nearly complete mitogenome of *L. boulardi* (Figitidae) is described. The main objective was to use this mitogenome to test the monophyly of the Cynipoidea superfamily and compare the synteny with *I. leucospoides* to verify whether the GO is a conserved character in Cynipoidea.

Specimens of *L. boulardi* were collected in Santa Maria, Brazil (latitude 34.95303 and longitude −120.43572). To collect the wasps, ripe bananas were placed in field sites for 4 days to allow oviposition. The fruits were then maintained in the laboratory until emergence of the flies and their parasitoid wasps (Ortiz *et al.*, 2015). Genomic DNA was isolated from a pool of 20 individuals using the NucleoSpin Tissue XS kit (Macherey-Nagel). The sample was sequenced using a Illumina HiSeq 2000 Next Generation Sequencing (NGS) device through the Fasteris DNA Sequencing Service (Plan-les-Ouates, Switzerland). A single-end approach with a read size of ~100 bp was employed. The reads were filtered by quality to eliminate low quality reads. The FASTX-Toolkit (http://hannonlab.cshl.edu/fastx_toolkit/) was implemented within the Galaxy webserver (Giardine *et al.*, 2005; Blankenberg *et al.*, 2010; Goecks *et al.*, 2010) using a quality cut-off value of 20 and a percent of bases that should possess a quality value equal to or higher than the cut-off value of 90 (Ortiz *et al.*, 2015). A total of 35,999,679 reads were selected as of high quality.

The mitogenome was assembled using the MITObim software package (Hahn *et al.*, 2013), with 102,482 reads being used in the assembly, this corresponding to 0.28% of total reads. The COI gene from *Lepitopila victoriae* (AB583620.1) was used as the seed for the assembly. MITObim uses an *in silico* baiting approach, which was implemented in the MIRAbait module of the MIRA assembler (v3.4.1.1) (Chevreux *et al.*, 1999). The *L. boulardi* mtDNA showed a coverage of 655.24 x (on average), and the test for circularity using the software mitoMaker showed it is not circularized. The sequence was deposited in GenBank under the accession number KU665622.

The characterization and annotation of the assembled *L. boulardi* mitogenome was performed on the MITOS Web Server (Bernt *et al.*, 2013a), using default parameters and UGENE software (Okonechnikov *et al.*, 2012), respectively.

The sequenced length of the genome was 15,417 bp, containing 13 protein-coding, two rRNA, and 21 tRNA genes, as well as an A+T-rich region having 316 bp and 84.2% of A+T content (Figure 1). We believe that a small portion of the genome failed to assemble because it was not possible to identify the trnM sequence. The mtDNA of *L. boulardi* is AT rich, totaling 80.3% A+T content. This high A+T composition is typical of other hymenopterans, with values ranging from 82.4% to 87.2% (Wei *et al.*, 2010). In the Hymenoptera species here studied, these values range from 77.7 to 87.4% (Supplementary Table S1). The plus strand comprised nine protein coding and two RNA genes, whereas the minus strand encompassed four protein coding genes and no RNA genes. All protein-coding genes initiated with an ATN codon, except for COI that started with the tetranucleotide, TTAG. Furthermore, among the protein-coding genes 11 had typical stop codons, TAA and TAG, while the ND6 and COII genes showed an incomplete stop codon T.

**Figure 1:**
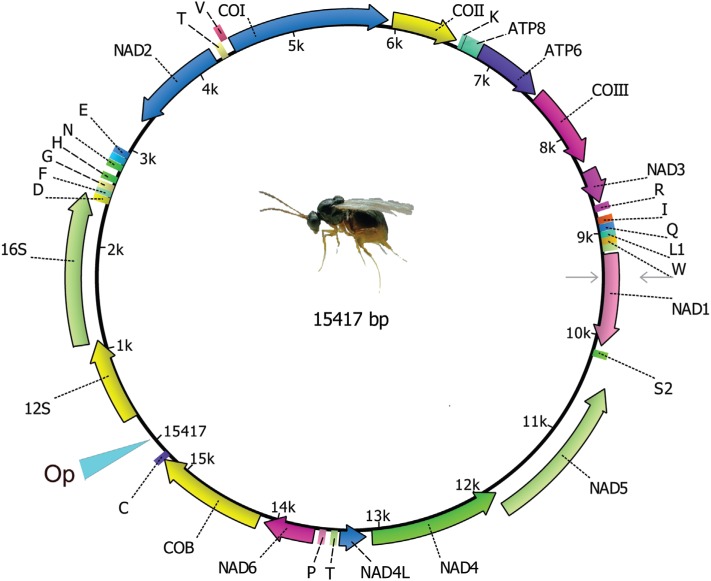
Summary of *L. boulardi* mitochondrial genome content and organization. ND1-6 and 4L refer to NADH dehydrogenase subunits 1-6 and 4L, COI-III refers to cytochrome c oxidase subunits 1-3, ATP6 and ATP8 refer to ATPase subunits 6 and 8, and Cyt b refers to cytochrome b; rrn refers to ribosomal RNA genes. Letters are the respective tRNAs genes. Op refers the missing region in the assemblage. Arrows indicate gene direction.

The synteny observed in the *L. boulardi* mitogenome differs from that found in PanGo (Figure 2). Two rearrangements involved protein-coding genes, Nad1 is here positioned between Nad3 and Nad5, Nad2 suffered an inversion, changing from the plus strand to the minus strand, and the rRNA genes rrnL and rrnS also underwent an inversion. An extensive change of positions was involved in the tRNAs genes. A total of 16 tRNA genes changed their positions compared with PanGo (trnL2, trnD, trnG, trnA, trnS1, trnN, trnE, trnF, trnH, trnS2, trnL1,trnV, trnI, trnQ, trnW, trnC) (Figure S1). Other five tRNA genes maintained synteny with respect to PanGo (trnK, trnR, trnT, trnP, trnY). As coverage is almost constant throughout the assembled genome, this indicates that the gene order is real. Looking at the full set of mitochondrial genes, an ample array of rearrangement was observed in *L. boulardi* when compared with the PanGo pattern, that is considered the ancestral gene order for insects.

**Figure 2:**
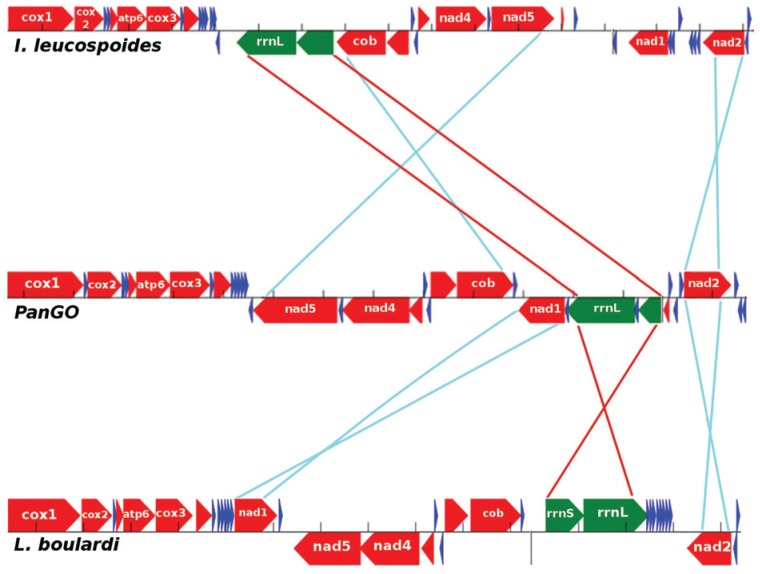
Rearrangements observed in the available mitogenomes of Cynipoidea compared to the Pancrustacea ancestral Gene Order (PanGo). In *Ibalia leucospoides,* the mitogenome has three large rearrangements, with a repositioning of seven protein-coding and two rRNA genes. Minor rearrangements also occcurred within the 15 tRNA genes (Mao *et al.*, 2015). In *L. boulardi*, there we found the presence of three large rearrangements that changed the position of two protein-coding and two rRNA genes. However, the rearrangements differ among the species.

Figure 2 also shows a synteny comparison of the *Ibalia leucospoides* mitochondrial genome with PanGo. The *Ibalia leucospoides* mitogenome showed extensive rearrangements involving 15 tRNA genes, as well as seven protein-coding genes compared to ancestral PanGo. Furthermore, it contains two extra trnM genes (Mao *et al.*, 2015). *I. leucospoides* and *L. boulardi* belong to the same superfamily, Cynipoidea, but are within different specific families. The first species is included in the Ibaliidae family and the second is a member of the Figitidae family. As can be observed in Figure 2, the rearrangements present in both genomes diverge significantly, suggesting that gene order is not conserved within the Cynipoidea and, thus, can be an important molecular marker for phylogenetic studies of this group, similar to other hymenopteran taxa (Oliveira *et al.*, 2008; Wei *et al.*, 2010; Mao *et al.*, 2015).

To address the question of the phylogenetic position of *L. boulardi,* eleven available complete mitogenomes were chosen as representatives of the main groups of Apocrita, as indicated by Sharkey (2007), Wei *et al.* (2010) and Sharkey *et al.* (2012). The species were: *Apis cerana* - Apoidea (NC_014295); *Cotesia vestalis* - Ichneumonoidea (NC_014272); *Diadegma semiclausum* -Ichneumonoidea (NC_012708); *Evania appendigaster* - Evanioidea (NC_013238); *Vespa mandarinia* - Vespoidea (NC_027172); *Megaphragma amalphitanum* - Chalcidoidea (NC_028196); *Orthogonalys pulchella* - Trigonaloidea (NC_025289); *Pelecinus polyturator* - Proctotrupoidea (NC_026865); *Philanthus triangulum* - Sphecoidea (NC_017007); and *Taeniogonalos taihorina* - Trigonaloidea (NC_027830); *Ibalia leucospoides-* Ibaliidae (NC_026832). The PanGO sequence, represented by *Drosophila incompta* (KM275233) was used as outgroup. These genomes were downloaded in January, 2016. The amino acid sequences of 13 coding genes of the mitogenomes were aligned separately using MUSCLE (Edgar, 2004) implemented in MEGA 5.0 (Tamura *et al.*, 2011), using default parameters. Subsequently the alignments were concatenated and trees were constructed using the maximum likelihood method, also in MEGA 5.0. The final alignment had a length of 3715 amino acid without gaps and 3875 with gaps. The evolutionary model employed was the aa-model mtRev (+F) and gamma distribution with invariant sites (G+I). Gap-missing data were treated as complete deletions. The support for each clade was measured with bootstrap values determined through the analysis of 500 pseudoreplicates.

The phylogenomic analysis positioned *L. boulardi* as the sister species of *I. leucospoides* (Figure 3). These species belong to the same superfamily, Cynipoidea, but belong to different families (Figitidae and Ibalidae respectively), and thus formed a monophyletic clade within the Apocrita phylogeny. The branches observed in the obtained tree are, in general, in accordance with the study of the evolutionary relationship among the Hymenoptera groups conducted by Sharkey *et al.* (2012). The major difference observed in the phylogenetic tree obtained here and that described by those authors is related to the group, Proctotrupomorpha, here represented by Cynipoidea, Proctotrupoidea, and Chalcidoidea. In the data of Sharkey *et al.* (2012), Cynipoidea clustered with Proctotrupoidea, and this being the sister group to Chalcidoidea. In the results obtained here, Cynipoidea clustered with Chalcidoidea, and this grouping with Proctotrupoidea. The other cluster retrieved in the phylogeny consists of Aculeata (Apoidea, Sphecoidea and Vespoidea), having as sister group the Evanioidea. This clade clustered with Trigonaloidea. The relationship seen for this clade is, thus, similar to that reported by Sharkey *et al.* (2012). Possible reasons for the differences observed between our results and those obtained by Sharkey *et al.* (2012) may be related to differences in the characters used for phylogenetic analysis. Sharkey *et al.* (2012) used 392 morphological characters and sequence data for four loci (18S, 28S, COI and EF-1a), while we used 13 mitochondrial genes.

**Figure 3:**
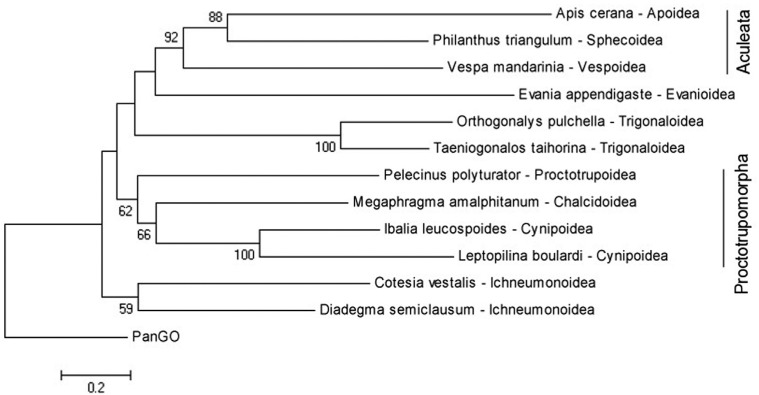
Maximum likelihood phylogenetic tree based on the amino acid sequences of 13 mitochondrial protein-coding genes for some representatives of the Aprocrita superfamilies. Genes were aligned separately and concatenated. The final alignment had a length of 3715 amino acid without gaps and 3875 with gaps. Trees were constructed using the aa-model mtRev (+F) and gamma distribution with invariant sites (G+I). Gap-missing data were treated as complete deletions. Bootstrap support is presented near each internal node.

In summary, we consider three main contributions of this study: i) the phylogenomic analyses showed that *L. boulardi* and *I. leucospoides* form a clade representative of the Cynipoidea superfamily within the Hymenoptera phylogeny, strengthening, as observed in other studies, the monophyly of the Cynipoidea superfamily; ii) the divergent gene order observed in *L. boulardi* and *I. leucospoides* suggests that this character is not conserved in Cynipoidea; and iii) the description of the *L. boulardi* partial mitochondrial genome should be relevant for future phylogenomics studies in Hymenoptera, as well being useful to future population genetics studies on this species.

## Supplementary Material

The following online material is available for this article:

Figure S1 - Rearrangements of tRNA genes between *Leptopilina boulardi* and PanGO.

Table S1 - Species used in this study, their superfamilies and families, accession numbers, data about the A+T region and total A+T content.
