# Supplementary material for: The rearranged mitochondrial genome of Leptopilina boulardi (Hymenoptera: Figitidae), a parasitoid wasp of Drosophila
Source: Genet Mol Biol. 2016 Sep 19;39(4):611–5. doi: 10.1590/1678-4685-GMB-2016-0062 (PMC5127158; doi:10.1590/1678-4685-GMB-2016-0062)
Supplement: Supplementary file 1 [file 1415-4757-gmb-1678-4685-GMB-2016-0062-Suppl01.pdf]

Table S1 - Species used in this study, their superfamilies and families, accession numbers, data about the A+T region and total A+T content.

Organism	Superfamily/Family	GenBank ID	A+T-rich region	Total A+T content
Leptopilina boulardi	Cynipoidea/Figitidae	KU665622	2977 to 3292 (316 bp-**84.2%**)	80.30%
Ibalia leucospoides	Cynipoidea/Ibaliidae	NC_026832	8090 to 8554 (464 bp-**83.3%**) and 16365 to 17212 (847 bp-**87.7%**)	86.30%
Apis cerana	Apoidea	NC_014295	15334 to 15895 (561 bp-**95.9%**)	83.90%
Cotesia vestalis	Ichneumonoidea/Braconidae	NC_014272	156 to 726 (570 bp-**92.7%**)	87.20%
Diadegma semiclausum	Ichneumoidea	NC_012708	16568 to 18728 (2160 bp-**96.4%**)	87.40%
Evania appendigaster	Evanioidea	NC_013238	15493 to 17817 (2324 bp-**85.6%**)	77.70%
Vespa mandarinia	Vespoidea	NC_027172	15703 to 15902 (199 bp-**88.5%**)	79.40%
Megaphragma amalphitanum	Chalcidoidea	NC_028196	Not described	85.30%
Orthogonalys pulchella	Trigonaloidea	NC_025289	15609 to 17277 (1668 bp-**88.8%**)	83.80%
Pelecinus polyturator	Proctotrupoidea	NC_026865	14705 to 14896 (191 bp-**95.3%**)	81.20%
Philanthus triangulum	Sphecoidea	NC_017007	14991 to 16029 (1038 bp-**85.7%**)	83.60%
Taeniogonalos taihorina	Trigonaloidea	NC_027830	4384 to 5304 (920 bp-**92.6%**)	84.60%
