# Supplementary material for: The rearranged mitochondrial genome of Leptopilina boulardi (Hymenoptera: Figitidae), a parasitoid wasp of Drosophila
Source: Genet Mol Biol. 2016 Sep 19;39(4):611–5. doi: 10.1590/1678-4685-GMB-2016-0062 (PMC5127158; doi:10.1590/1678-4685-GMB-2016-0062)
Supplement: Supplementary file 2 [file 1415-4757-gmb-1678-4685-GMB-2016-0062-Suppl02.pdf]

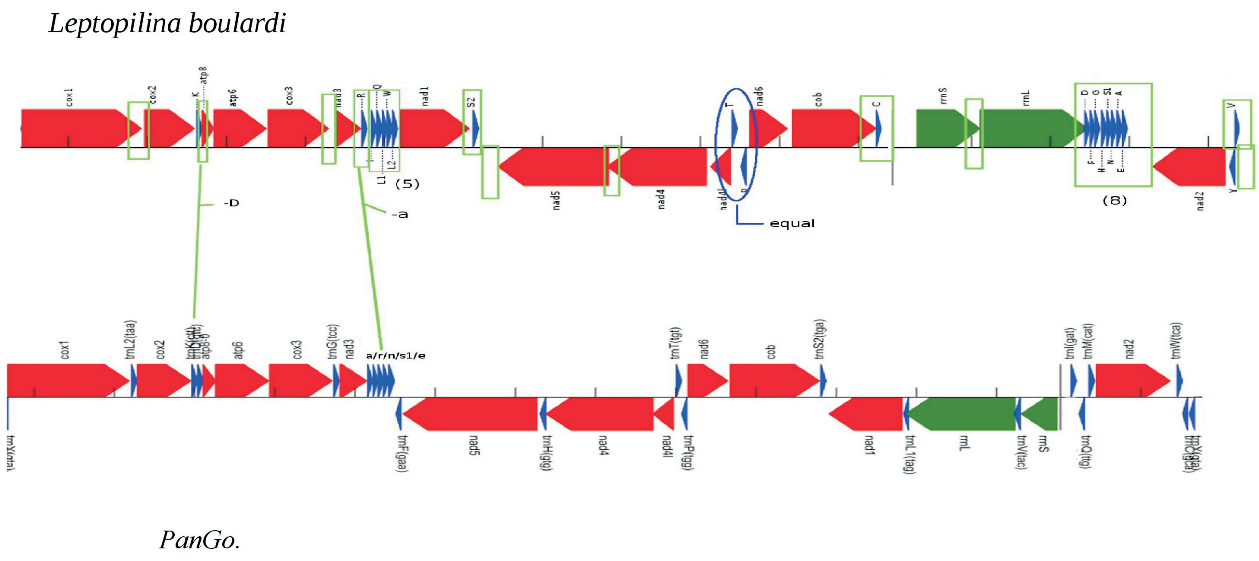

tRNA gene that have maintained position in relation to PanGo: trnK, trnR, trnT, trnP, trnY.

and trnL2, trnD, trnG, trnA, trnS1, trnN, trnE, trnF, trnH, trnS2, trnL1, trnV, trnI, trnQ, trnW, trnC, have moved for other positions.

Figure S1 - Rearrangements of tRNA genes between *Leptopilina boulardi* and PanGO.
